# Modulation of osteogenic differentiation by *Escherichia coli*-derived recombinant bone morphogenetic protein-2

**DOI:** 10.1186/s13568-022-01443-5

**Published:** 2022-08-10

**Authors:** Nam-Hyun Kim, Seon-Kyong Jung, Juno Lee, Pahn-Shick Chang, Seung-Hoon Kang

**Affiliations:** 1 Life Science Institute, Daewoong Pharmaceutical, Yongin, Gyeonggido Republic of Korea; 2 Department of Agricultural Biotechnology, Seoul National University, Seoul, Republic of Korea; 3 Research Institute of Agriculture and Life Sciences, Seoul National University, Seoul, Republic of Korea; 4 Center for Food and Bioconvergence, Seoul National University, Seoul, Republic of Korea; 5 Center for Agricultural Microorganism and Enzyme, Seoul National University, Seoul, Republic of Korea

**Keywords:** rhBMP-2, Osteogenesis, BMP receptor, Smad-signaling pathway, qRT-PCR, ALP assay

## Abstract

Recombinant human bone morphogenetic protein-2 (rhBMP-2), a key regulator of osteogenesis, induces the differentiation of mesenchymal cells into cartilage or bone tissues. Early orthopedic and dental studies often used mammalian cell-derived rhBMP-2, especially Chinese hamster ovary (CHO) cells. However, CHO cell-derived rhBMP-2 (C-rhBMP-2) presents disadvantages such as high cost and low production yield. To overcome these problems, *Escherichia coli*-derived BMP-2 (E-rhBMP-2) was developed; however, the E-rhBMP-2-induced signaling pathways and gene expression profiles during osteogenesis remain unclear. Here, we investigated the E-rhBMP-2-induced osteogenic differentiation pattern in C2C12 cells and elucidated the difference in biological characteristics between E-rhBMP-2 and C-rhBMP-2 via surface plasmon resonance, western blotting, qRT-PCR, RNA-seq, and alkaline phosphatase assays. The binding affinities of E-rhBMP-2 and C-rhBMP-2 towards BMP receptors were similar, both being confirmed at the nanomolecular level. However, the phosphorylation of Smad1/5/9 at 3 h after treatment with E-rhBMP-2 was significantly lower than that on treatment with C-rhBMP-2. The expression profiles of osteogenic marker genes were similar in both the E-rhBMP-2 and C-rhBMP-2 groups, but the gene expression level in the E-rhBMP-2 group was lower than that in the C-rhBMP-2 group at each time point. Taken together, our results suggest that the osteogenic signaling pathways induced by E-rhBMP-2 and C-rhBMP-2 both follow the general Smad-signaling pathway, but the difference in intracellular phosphorylation intensity results in distinguishable transcription profiles on osteogenic marker genes and biological activities of each rhBMP-2. These findings provide an extensive understanding of the biological properties of E-rhBMP-2 and the signaling pathways during osteogenic differentiation.

## Introduction

Urist and Cols were the first to identify the phenomenon of bone formation by auto-induction in a mineralized bone matrix in 1965; they named the matrix glycoprotein that induced the differentiation of mesenchymal cells into bone marrow cells and bone as bone morphogenetic protein (BMP) (Urist 1965; Urist and Strates 1971). Various studies on the isolation and purification of BMPs followed, and BMP-2 was the first to be derived from mammalian cells via cloning in 1988 (Wozney et al. 1988). Analyses of cDNA and amino acid sequences identified BMP-2 as a protein that belongs to the transforming growth factor-β (TGF-β) superfamily, whose proteins show biological activity in homo-dimeric form when binding to type I and type II serine-threonine kinase receptors (BMP receptor type 1 and 2, respectively) (Quaas et al. 2018).

Recombinant human BMP-2 (rhBMP-2) induces the differentiation of mesenchymal cells into cartilage or bone tissues and is a key regulator of osteogenesis (Kirker-Head 2000). Various clinical applications utilizing the functions of rhBMP-2 have been attempted in orthopedics and dentistry (Namikawa et al. 2005). The most widely recognized commercial product based on rhBMP-2 is Infuse™, which has already received FDA approval (Ong and Bouazza-Marouf 2000; Even et al. 2012).

Early studies often used mammalian cells-derived rhBMP-2, especially from Chinese hamster ovary cells (C-rhBMP-2). However, C-rhBMP-2 presents disadvantages such as high cost of culture and purification as well as low production yield. Some studies have attempted to address these disadvantages by producing rhBMP-2 in *Escherichia coli* to achieve high production and were able to refold rhBMP-2 in an active dimeric form through in vitro renaturation (Ruppert et al. 1996; Kübler et al. 1998). The most notable difference between C-rhBMP-2 and *E. coli*-derived rhBMP-2 (E-rhBMP-2) is the presence or absence of post-translational glycosylation. Proteins produced from Chinese hamster ovary (CHO) cells contain glycans in their final expressed form, whereas proteins produced from *E. coli* do not contain glycans. Various studies have reported that E-rhBMP-2 exhibits biological activities similar to those of C-rhBMP-2, despite structural differences. According to these studies, C-rhBMP-2 has a higher in vitro potency but similar in vivo efficacy compared to that of E-rhBMP-2 (Fung et al. 2019; Kim et al. 2011; Suzuki et al. 2020).

The BMP-2 signaling pathway starts with the BMP-2 ligand forming a complex with BMP type I and II receptors on the cell surface. Subsequently, the serine/threonine kinase of the BMP type I receptor is activated and intracellular receptor-regulated Smads (R-Smads) are phosphorylated. Phosphorylated R-Smad subsequently forms a complex with the common mediator Smad, Smad4. The complex is then transferred into the nucleus and initiates the expression of transcription factors that induce differentiation into osteocytes and chondrocytes (Wang et al. 2014; Pasero et al. 2012; Miyazono et al. 2010; Prashar et al. 2014). Moreover, it has been reported that E-rhBMP-2 may have the same signaling pathway as C-rhBMP-2, based on a phosphorylation assay of R-Smads and osteogenic gene expression studies (Yano et al. 2009). Recently, whole genome RNA sequencing (RNA-seq) based on high-throughput next-generation sequencing (NGS) technology was applied to identify the transcriptomes associated with osteogenesis (Han et al. 2018; Shigemizu et al. 2020; Shaik et al. 2019; Zhou et al. 2021). Additionally, the rhBMP-2-dependent gene regulatory network was analyzed by RNA-seq to characterize the role of differentially expressed transcription factors on osteoblast differentiation, but the difference induced by E-rhBMP-2 and C-rhBMP-2 in biological activity and osteogenic gene expression profile during whole in vitro osteogenesis remains unclear (Yu et al. 2021; Wei et al. 2020).

This study aimed to provide information on the characteristics of E-rhBMP-2 during osteogenic differentiation in an in vitro system compared with the characteristics of C-rhBMP-2. Herein, the signaling pathway induced by E-rhBMP-2 was investigated and the equilibrium dissociation rate constants (*K*_*D*_) with three types of BMP-2 receptors were determined. The phosphorylation profile of R-Smad 1/5/9 in the cytoplasm and the expression profile of osteogenic marker genes were also evaluated. Moreover, we conducted an in vitro comparison of alkaline phosphatase (ALP) expression levels between E-rhBMP-2 and C-rhBMP-2, which are representative osteogenic marker proteins. Our results provide information on the osteogenic signaling pathway and biological characteristics induced by E-rhBMP-2.

## Materials and methods

### Preparation of rhBMP-2

C-rhBMP-2 was purchased from National Institute for Biological Standards and Control (Hertfordshire, UK). The C-rhBMP-2 was prepared by reconstitution with 1 mL of distilled water in accordance with the manufacturer’s protocol. E-rhBMP-2, a homo-dimeric protein with a mature rhBMP-2 sequence, was provided by Daewoong Pharmaceutical Co., Ltd. (Seoul, Republic of Korea). The E-rhBMP-2 was prepared in a buffer containing 25 mg/mL glycine, 3.7 mg/mL glutamic acid, 5 mg/mL sucrose, 0.1 mg/mL NaCl, and 0.1 mg/mL polysorbate 80.

### Surface plasmon resonance analysis

Surface plasmon resonance (SPR) measurements were performed using a Biacore T200 instrument (GE Healthcare, Pittsburgh, PA, USA). BMP receptor type 1A (BMPR1A), BMP receptor type 1B (BMPR1B), and BMP receptor type 2 (BMPR2) were purchased from Thermo Fisher Scientific Co. (Rockford, IL, USA). A mixture of 0.4 M 1-ethyl-3-(3-dimethylaminopropyl) carbodiimide and 0.1 M N-hydroxysuccinimide was injected over both the sample (E-rhBMP-2 and C-rhBMP-2) and reference (BMPR1A, BMPR1B, and BMPR2) channel for 7 min at a flow rate of 10 µL/min.

The immobilization target level was set to 500 RU for each ligand (E-rhBMP-2 and C-rhBMP-2). E-rhBMP-2 stock samples and C-rhBMP-2 stock samples were diluted to 5.9 µg/mL and 0.5 µg/mL, respectively, in 10 mM sodium acetate (pH 6.0) buffer. The prepared ligand samples were only injected and immobilized on the sample channel. To deactivate the surface, 1 M ethanolamine (pH 8.5) was injected into both the sample and reference channels for 7 min at 10 µL/min.

All analyte samples (BMPR1A, BMPR1B, and BMPR2) were diluted in a concentration range of 10–2000 nM. Samples were injected into the sample and reference channels at a flow rate of 30 µL/min for an association phase of 120 s, followed by 120 s of dissociation to determine the association time, dissociation time, and concentration range of analysis. To test the regeneration conditions, NaOH in the concentration range of 10–50 mM was injected for 10 s at a flow rate of 30 µL/min. A 1:1 binding model describing one molecule of analyte binding to a single ligand molecule was used to calculate the *K*_*D*_.

### C2C12 cell culture

Myoblastic C2C12 cells were purchased from the American Type Culture Collection (Rockville, MD, USA). C2C12 cells were grown in Dulbecco’s modified Eagle’s medium (DMEM) containing 10% fetal bovine serum (FBS) and 1% penicillin–streptomycin (PS) at 37 °C and 5% CO_2_ humidified air using T75 flasks. When the cells reached confluence, they were used for other experiments. All reagents for cell culture were purchased from Gibco™ (Langley, VA, USA).

### Western blot analysis

Cultured C2C12 cells were collected and seeded in 6-well plates (2 × 10^5^ cells/well). The plates were then incubated at 37 °C and 5% CO_2_ for 24 h. E-rhBMP-2 and C-rhBMP-2 solutions were prepared at a concentration of 100 ng/mL by diluting with DMEM containing 2% bovine serum (BS). The prepared solutions were added to each well of a 6-well plate and incubated at 37 °C and 5% CO_2_. The cells were collected at 0, 3, and 6 h after rhBMP-2 treatment. The collected cells were lysed using a lysis buffer (50 mM Tris–HCl (pH 6.8), 2% SDS, 0.001% bromophenol blue, and 10% glycerol). The cell lysate was centrifuged (12,000×*g*) at 4 °C, after which the supernatant was recovered and protein concentration in the supernatant was measured using a BCA protein assay kit (Thermo Fisher Scientific Co) according to manufacturer instructions. Electrophoresis was carried out at 200 V for 30 min after loading 200 μg of protein into each well of 4–12% Bis–Tris Gels (Invitrogen, Carlsbad, CA, USA). The separated proteins from the gel were then transferred onto PVDF membranes (Thermo Fisher Scientific Co.). The membranes were blocked for 1 h with 5% skim milk, followed by reaction with anti-Phospho-Smad1/Smad5/Smad9 rabbit antibody (Danvers, MA, USA) diluted 1:1000 at 4 °C for 24 h. The membranes were then incubated with anti-rabbit HRP secondary antibody (Cambridge, MA, USA) diluted 1:2000 at 25 °C for 2 h. Peroxidase activity on the PVDF membrane was visualized by adding 3,3′,5,5′-Tetramethylbenzidine. Bands were detected after color development using a digital imaging system (Amersham Imager 600, GE Healthcare, Pittsburgh, PA, USA). The sampling process was repeated using primary antibodies against Smad1 and β-actin (Thermo Fisher Scientific Co.). Quantitation of the western blot was performed using GelAnalyzer 2010a software (http://www.gelanalyzer.com).

### qRT-PCR analysis

Cultured C2C12 cells were collected, and 2 mL of the cell solution (2 × 10^5^ cells/mL) was seeded in 6-well plates. The cells were incubated at 37 °C and 5% CO_2_ for 24 h. E-rhBMP-2 and C-rhBMP-2 solutions were prepared at a concentration of 1000 ng/mL by diluting with DMEM containing 2% BS. After treating the cells with the prepared rhBMP-2 solutions, they were incubated at 37 °C and 5% CO_2_ and recovered after 18 and 24 h of incubation. After using the PureLink™ RNA mini kit (Invitrogen) to extract mRNA from the collected cells, a High-Capacity RNA-to-cDNA™ kit (Thermo Fisher Scientific Co.) was used to obtain cDNA.

The primers for osteogenic genes (Runt-related transcription factor 2 (*Runx2*) and osteocalcin (*OCN*)) and β-actin gene (internal control) were purchased from Thermo Fisher Scientific Co. (*Runx2*: Mm00501580_m1, *OCN*: Mm03413826_mH, *β-actin*: Mm00607939_s1). PCR was performed using TaqMan™ Fast Advanced Master Mix (Applied Biosystems, Foster City, CA, USA), and the expression levels of *Runx2* and *OCN* genes relative to *β-actin* were measured according to the manufacturer's instructions. For qRT-PCR analysis, experiments were performed in triplicate and values were expressed as the mean ± standard deviation (SD). Minitab 14 (Minitab Ltd, USA) was used to perform statistical analyses. Student's *t*-test was used to analyze differences in the expression level of the *Runx2* and *OCN* genes between E-rhBMP-2 and C-rhBMP-2 at each time point. A value of *P* < 0.05 was considered significant.

### ALP assay

Cultured C2C12 cells were collected, and the cell suspension was diluted with culture media to reach a concentration of 1 × 10^5^ cells/mL. After adding 100 µL of the C2C12 cell solution (1 × 10^5^ cells/mL) to each well of a 96-well plate, the cells were fixed by incubation at 37 °C and 5% CO_2_ for 24 h. E-rhBMP-2 and C-rhBMP-2 were prepared to a concentration of 5–1000 ng/mL (5.0, 23.3, 37.3, 59.6, 95.4, 152.6, 244.1, 390.6, 625.0, and 1000.0 ng/mL) by diluting with DMEM containing 2% BS. The prepared rhBMP-2 solutions at each concentration were used for treating the C2C12 cell solutions in triplicate in a 96-well plate. After 72 h of incubation, all solutions in the 96-well plate were removed and 100 µL of lysis buffer (100 mM glycine, 1 mM magnesium chloride, 1 mM zinc chloride, and 1% tergitol, pH 9.6) was added to each well to lyse the cells. Upon completion of lysis, 50 µL of *p*-nitrophenyl phosphate was added to each well followed by incubation at 25 °C for 30 min. Upon completion of the reaction, the absorbance at 405 nm was measured using a SpectraMax^®^ M3 microplate reader (Molecular Devices LLC, Sunnyvale, CA, USA). Parallel-line assays were performed based on the measured dose–response curves using PLA 2.0 (Stegmann Systems GmbH, Germany). The experiments were performed on three separate plates with three replicates each.

### Library construction and sequencing

Cultured C2C12 cells were collected and seeded in 6-well plates (2 × 10^5^ cells/well). The plates were incubated at 37 °C and 5% CO_2_ for 24 h. E-rhBMP-2 and C-rhBMP-2 solutions were prepared at a concentration of 100 ng/mL by diluting with DMEM containing 2% BS. After treating each well of the 6-well plate with the prepared solutions, the plate was incubated at 37 °C and 5% CO_2_. The cells were collected after 3, 6, 12, and 24 h of incubation and mRNA was extracted as described in the qRT-PCR analysis section.

The acquired mRNA was purified using the Dynabeads® mRNA Purification Kit (Invitrogen) to deplete rRNA and enrich poly(A) + RNA using oligo d(T). Enriched mRNA was used for library construction using the MGIEasy RNA Directional Library Prep Kit (MGI, Shenzhen, China), according to the manufacturer’s instructions. The directional RNA libraries were then sequenced using the DNBSEQ-T7 sequencing instrument (BGI Genomics, China), according to the manufacturer’s instructions, yielding 150 bp paired-end reads. Sequencing results from this study have been deposited in the NCBI Short Read Archive under the accession number PRJNA812069.

### Sequence alignment and gene expression analysis

Adapter sequence and low-quality bases were removed using the Cutadapt tool (version 2.9) (Martin 2011) and sequences under 36 bp were discarded by using the Trimmomatic tool (version 0.39) (Bolger et al. 2014). After trimming, reads were aligned to the mouse reference genome (mm10) and annotated by Ensembl v.102 using STAR (version 2.7.3a) with default parameters (Dobin et al. 2012). The read count and transcript per million values for individual transcripts were produced using the RSEM (version 1.3.1) software tool with default parameters (Li and Dewey 2011). The Bioconductor package edgeR was applied to identify differentially expressed genes (DEGs) with a *P*-value of 0.05 and fold change ≥ 2 criteria (Robinson et al. 2010).

## Results

### Binding affinity of rhBMP-2/BMP receptor

SPR experiments were performed using Biacore T200 optical biosensors to determine the binding affinity between E-rhBMP-2 and BMP receptors. The resulting data were fitted to a 1:1 binding kinetic model using BiaEvaluation software v3.2. According to the results of the 1:1 binding model in E-rhBMP-2 (Table 1), *K*_*D*_ of BMPR1A, BMPR1B, and BMPR2 was 1.52 × 10^–8^ M, 1.94 × 10^–7^ M, and 1.40 × 10^–7^ M, respectively. Likewise, the *K*_*D*_ between C-rhBMP-2 and BMPR1A, BMPR1B, and BMPR2 were 1.09 × 10^–8^ M, 7.43 × 10^–8^ M, and 1.41 × 10^–7^ M, respectively. E-rhBMP-2 preferably bound, in the following order, to BMPR1A, BMPR2, and BMPR1B. In the case of C-rhBMP-2, the protein preferably bound, in the following order, to BMPR1A, BMPR1B, and BMPR2. The binding affinity between BMPR1A and rhBMP-2 was the highest with E-rhBMP-2 and C-rhBMP-2 both.

**Table 1:** Binding kinetic parameters of E-rhBMP-2 and C-rhBMP-2

Ligand	Analyte	*k*_*a*_ (L/Ms)	*k*_*d*_ (L/s)	*K*_*D*_ (M)	R_max_ (RU)
E-rhBMP-2	BMPR1A	1.03 × 10^4^	1.57 × 10^–4^	1.52 × 10^–8^	223.2
	BMPR1B	1.96 × 10^3^	3.80 × 10^–4^	1.94 × 10^–7^	43.6
	BMPR2	6.07 × 10^3^	8.47 × 10^–4^	1.40 × 10^–7^	107.2
C-rhBMP-2	BMPR1A	1.18 × 10^4^	1.29 × 10^–4^	1.09 × 10^–8^	347.5
	BMPR1B	3.47 × 10^3^	2.58 × 10^–4^	7.43 × 10^–8^	109.9
	BMPR2	4.85 × 10^3^	6.86 × 10^–4^	1.41 × 10^–7^	222.2

*k*_*a*_, association rate; *k*_*d*_, dissociation rate; *K*_*D*_, equilibrium dissociation rate constant; R_max_, analyte binding capacity of the surface

### rhBMP-2-induced Smad phosphorylation

**Fig. 1:**
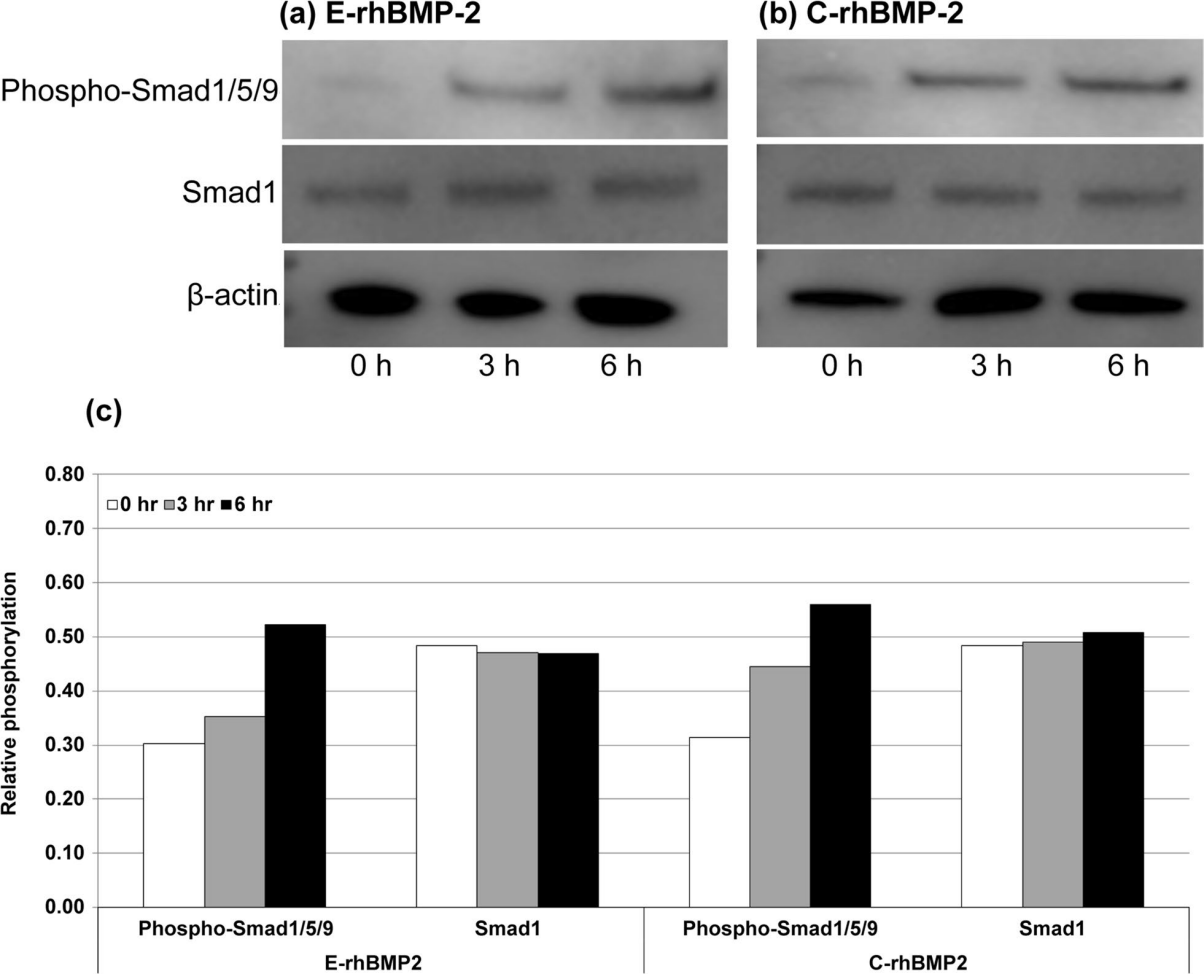
Effects of E-rhBMP-2 and C-rhBMP-2 on intracellular receptor-regulated Smads in C2C12 cells. A total of 0.2 mg protein was loaded onto each lane and anti-phospho-Smad1/5/9 antibody was applied to detect the phospho-Smad1/5/9. Bands for Smad1 and β-actin are shown as the loading control. Bands were detected using Amersham Imager 600 (GE Healthcare). **a** E-rhBMP-2, *Escherichia coli*-derived recombinant human bone morphogenetic protein-2; **b** C-rhBMP-2, rhBMP-2 derived from CHO cell; **c** Relative phosphorylation was calculated normalizing the quantification values of phospho-Smad1/5/9 and Smad1 band intensity to the β-actin band intensity

Western blotting was performed to confirm the phosphorylation of Smads (Smad1/5/9), which are signal transducers regulated by rhBMP-2 and BMP receptors. The results confirmed that treatment with rhBMP-2 induced the phosphorylation of Smad1/5/9 in C2C12 cells (Fig. 1). The Smad1/5/9 phosphorylation tendency gradually increased up to 6 h for both E-rhBMP-2 and C-rhBMP-2. However, the induced phosphorylation of Smad1/5/9 at 3 h after rhBMP-2 treatment was weaker in E-rhBMP-2 than in C-rhBMP-2. Smad1 and β-actin, which were used as controls, showed constant expression regardless of the types of rhBMP-2 used and induction time.

### Effects of rhBMP-2 on Runx2 and OCN expression

**Fig. 2:**
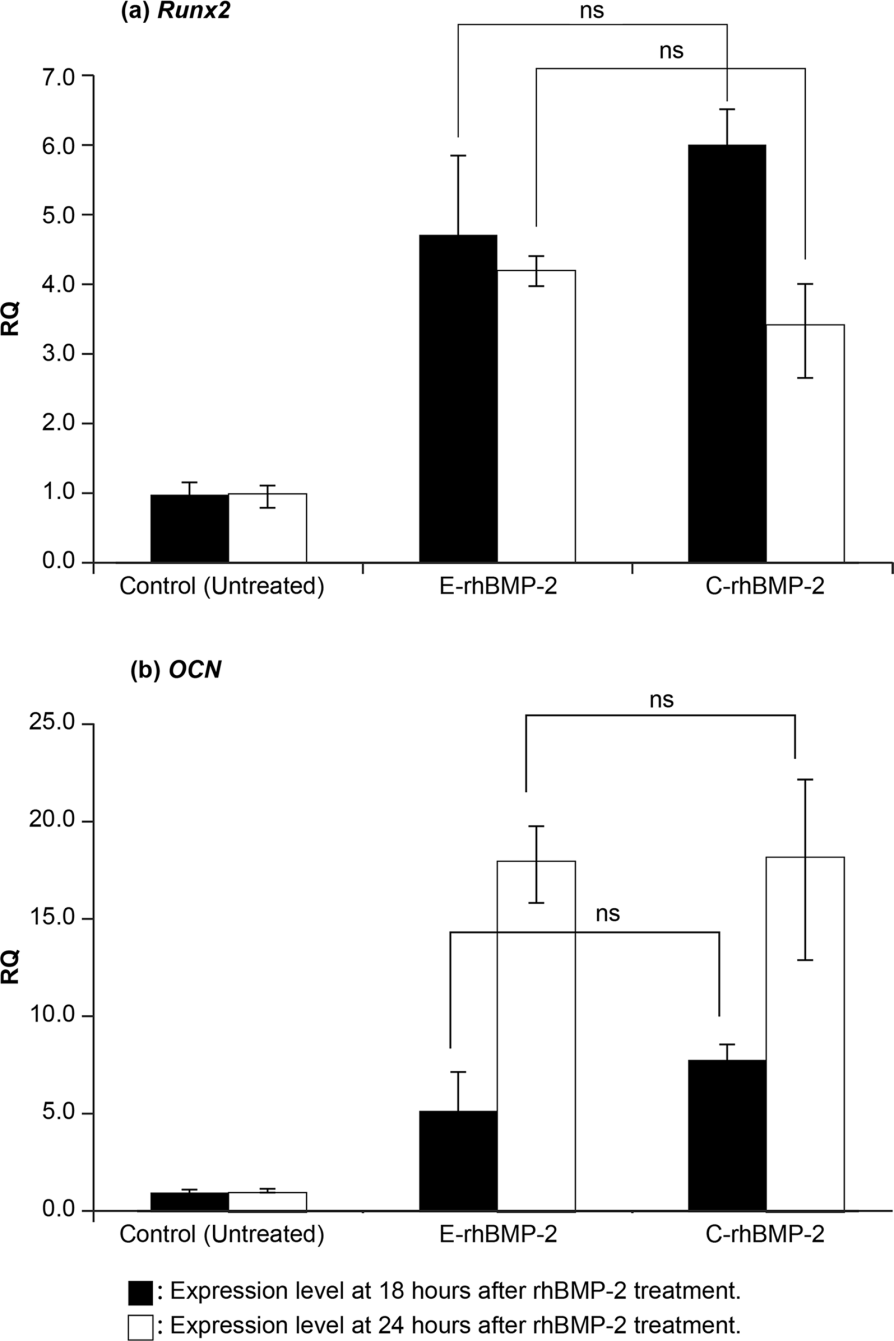
Relative mRNA expression levels of *Runx2* and *OCN* in rhBMP-2-treated C2C12 cells at 18 h and 24 h. **a** Relative expression level of *Runx2*, **b** relative expression level of *OCN*

The mRNA expression levels of commonly-used marker genes, *Runx2* and *OCN*, were identified in C2C12 cells using qRT-PCR to investigate the effects of E-rhBMP-2 and C-rhBMP-2 on osteoblastic differentiation. The relative expression levels of *Runx2* and *OCN* genes at 18 h and 24 h were increased in both the E-rhBMP-2 and C-rhBMP-2 groups compared to those in the control. There was no significant difference in the expression levels of *Runx2* and *OCN* genes between the E-rhBMP-2 and C-rhBMP-2 groups at the same treatment times (Fig. 2). In both the E-rhBMP-2 and C-rhBMP-2 groups, the relative expression level of the *Runx2* gene decreased with time, and the reduction of *Runx2* expression level over time was smaller in E-rhBMP-2 than in C-rhBMP-2. On the contrary, the relative expression level of the *OCN* gene increased with time in both the E-rhBMP-2 and C-rhBMP-2 groups, and expression levels of the *OCN* gene at 24 h were similar in both E-rhBMP-2 and C-rhBMP-2 groups.

### Effects of rhBMP-2 on ALP activity

**Fig. 3:**
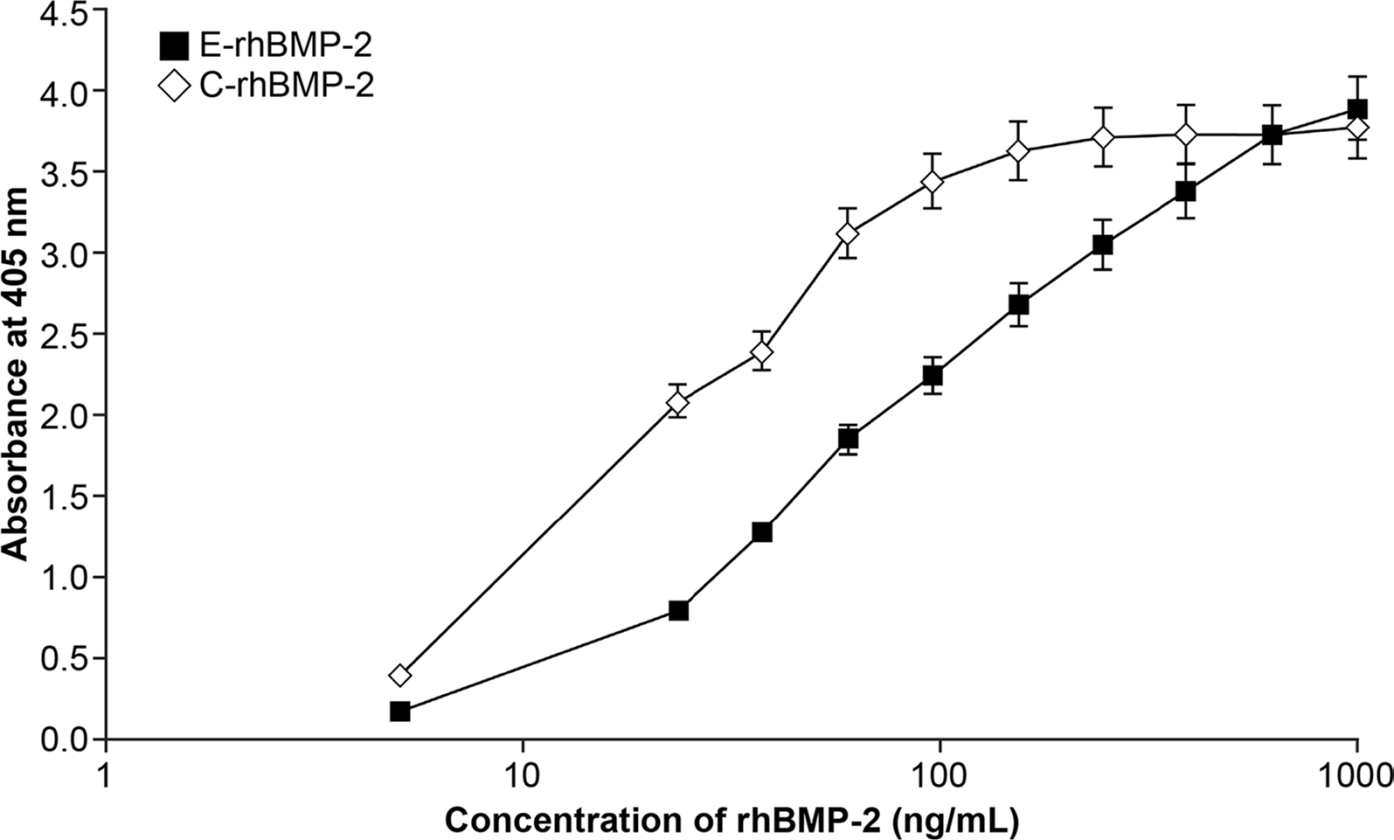
Effect of E-rhBMP-2 and C-rhBMP-2 concentration on ALP activity in C2C12 cells. Optical density values were recorded at 405 nm after 72 h of culture

ALP is a typical osteogenic marker known to be expressed during osteoblastic differentiation. The ALP activity of C2C12 cells was measured according to the concentrations of E-rhBMP-2 and C-rhBMP-2 after 72 h. The ALP activity of C2C12 cells increased in a dose-dependent manner from 10 to 500 ng/mL with an increase in the concentration of E-rhBMP-2 and C-rhBMP-2 (Fig. 3). The EC_50_ values of E-rhBMP-2 and C-rhBMP-2 determined from 4-parameter curves based on a total of three assays were 78.6 ± 6.69 ng/mL and 22.1 ± 2.55 ng/mL, respectively. Moreover, the potency of E-rhBMP-2 relative to that of C-rhBMP-2 determined by parallel line assay was 0.306 ± 0.02 (Table 2).

**Table 2:** Comparison of in vitro biological activity between E-rhBMP-2 and C-rhBMP-2

E-rhBMP-2		C-rhBMP-2		Relative potency of E-rhBMP-2 based on C-rhBMP-2
Slope	EC_50_^a^ (ng/mL)	Slope	EC_50_^a^ (ng/mL)	
0.93 ± 0.14	78.6 ± 6.69	1.46 ± 0.13	22.1 ± 2.55	0.306 ± 0.02

^a^EC_50_, half-maximal effective concentration, values were calculated by analyzing the results using a 4-parameter curve

### Overall gene expression profile induced by rhBMP-2

RNA-seq analysis was performed for a more in-depth understanding of the overall gene expression following E-rhBMP-2 and C-rhBMP-2 treatment. The RNA-seq data were analyzed in two ways. First, DEG analysis was performed to compare the gene profile of C2C12 cells between E-rhBMP-2 and C-rhBMP-2 treatment for each time. Out of the 21,868 genes in total, only two DEGs between E-rhBMP-2 and C-rhBMP-2 were identified at 3, 6, and 12 h. The number of DEGs rapidly increased to 85 at 24 h, including 11 up-regulated genes in E-rhBMP-2 and 74 up-regulated genes in C-rhBMP-2 (Table 3).

**Table 3:** Differentially expressed genes between E-rhBMP-2 and C-rhBMP-2 in C2C12 cells

Incubation time	E-rhBMP-2 Up-regulated gene counts	C-rhBMP-2 Up-regulated gene counts	Total DEG	DEG ratio of total gene counts (%)^a^
3 h	2	0	2	0.009
6 h	1	1	2	0.009
12 h	1	1	2	0.009
24 h	11	74	85	0.389

DEG, differentially expressed genes

^a^DEG ratio was presented as percentage of total DEGs out of the total gene counts (21,868) at each time point

Next, the expressions of osteogenic marker gene in C2C12 cells not treated with rhBMP-2 (control) and that of C2C12 cells treated with C-rhBMP-2 or E-rhBMP-2 were compared. Significant changes were observed in expression levels of the nine osteogenic marker genes at 24 h after E-rhBMP-2 and C-rhBMP-2 treatment (Table 4). The osteogenic marker genes showed a similar profile in both the E-rhBMP-2 and C-rhBMP-2 groups. However, the expression level of each gene was lower under E-rhBMP-2 treatment than C-rhBMP-2 treatment. Moreover, the expression levels of nine osteogenic marker genes at 24 h after C-rhBMP-2 treatment were significantly different compared to those in the control, whereas the expression levels of three genes (TGF-β-inducible early gene (*TIEG1*), *Runx2*, and *OPG*) at 24 h after E-rhBMP-2 treatment were not significantly different.

**Table 4:** Expression levels of osteogenic marker genes at 24 h after rhBMP-2 treatment

Gene		E-rhBMP-2		C-rhBMP-2	
Abbreviation	Full name	Log_2_FC	*P*-value	Log_2_FC	*P*-value
*Oasis*	Old astrocyte specifically induced substance	1.29	9.64 × 10^–9^	3.20	1.70 × 10^–66^
*TIEG1*	TGF-β-inducible early gene	0.84	2.08 × 10^–2^	1.37	3.71 × 10^–5^
*Id3*	Inhibitor of DNA Binding 3	1.60	1.53 × 10^–27^	1.69	1.00 × 10^–30^
*Runx2*	BMP-2-induced runt-related transcription factor 2	0.70	7.99 × 10^–2^	1.83	1.81 × 10^–8^
*Col1*	Type 1 collagen	2.60	2.88 × 10^–21^	4.38	1.34 × 10^–84^
*OSX*	Osterix	6.12	8.45 × 10^–15^	7.77	2.33 × 10^–40^
*OPG*	Osteoprotegerin	3.21	5.01 × 10^–1^	7.11	2.28 × 10^–10^
*OPN*	Osteopontin	− 1.39	2.08 × 10^–32^	-2.93	7.33 × 10^–108^
*OCN*	Osteocalcin	4.96	2.06 × 10^–3^	7.54	2.14 × 10^–16^

**Fig. 4:**
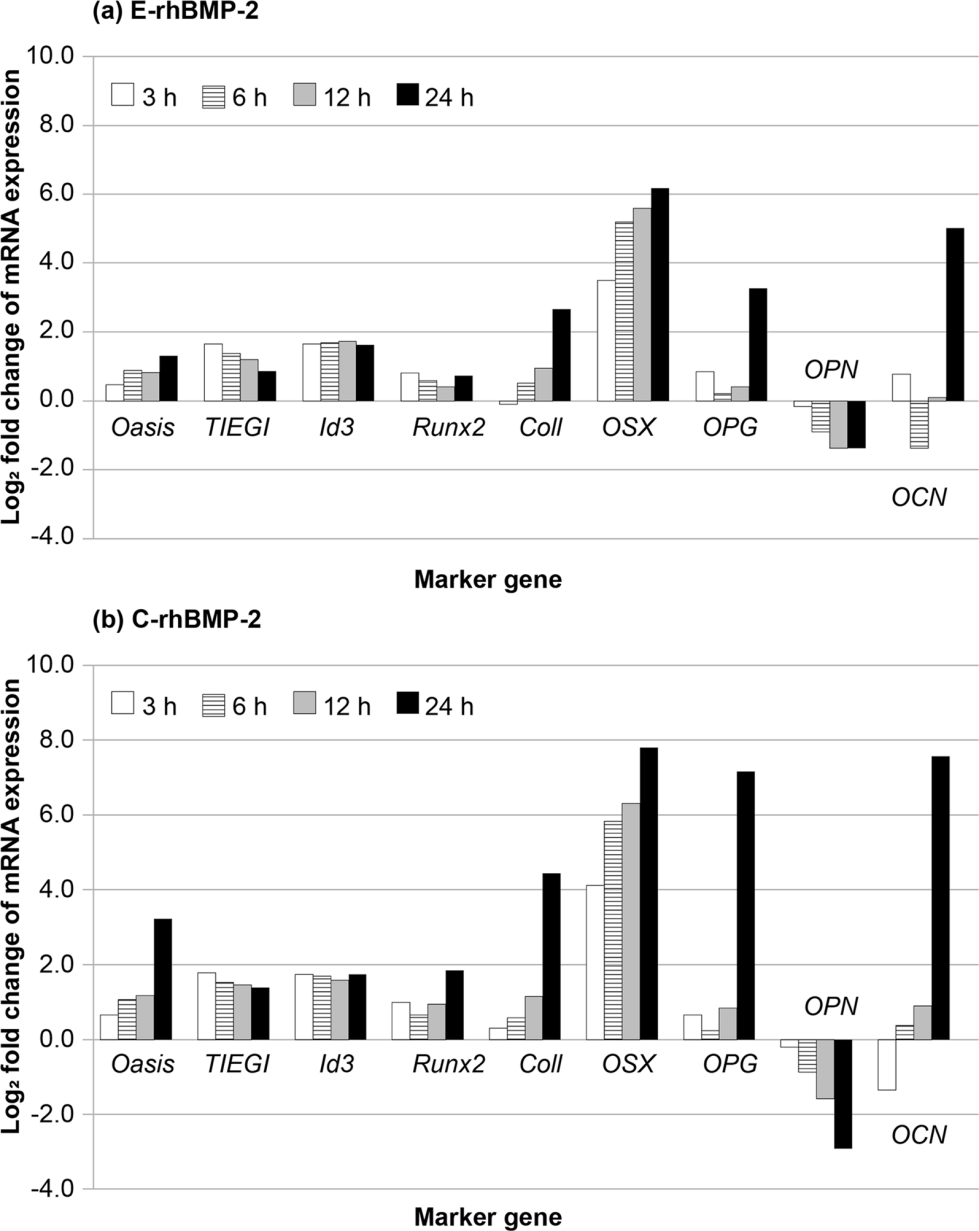
Time-series expression profile of osteogenic marker genes after rhBMP-2 treatment. **a** E-rhBMP-2, **b** C-rhBMP-2

The expression profiles of nine osteogenic marker genes at 3–24 h after rhBMP-2 treatment were similar to those of C-rhBMP-2 treatment (Fig. 4). Changes in the expression levels of immediate early response genes were similar between the E-rhBMP-2 and C-rhBMP-2 groups, whereas the expression level of the old astrocyte specifically induced substance (*OASIS*) gene after 24 h was approximately 4 times higher under treatment with C-rhBMP-2 than under E-rhBMP-2 treatment.

## Discussion

This study aimed to characterize the biological properties and signaling pathways of *E. coli*-derived rhBMP-2 during osteogenesis. The study used SPR analysis, western blot assays, qRT-PCR, RNA-seq analysis, and ALP assays to analyze and identify the binding affinity between each rhBMP-2 and BMP receptors on the cell surface (BMPR1A, BMPR1B, and BMPR2), subsequent intracellular phosphorylation, expression profiles of osteogenic genes in the nucleus, and ALP (differentiated osteoblast marker) activity.

The major structural difference between E-rhBMP-2 and C-rhBMP-2 is the absence or presence of an N-glycan on the protein surface. However, the absence of N-glycan on E-rhBMP-2 demonstrates little or no impact on binding with BMP receptors, since the *K*_*D*_ values of each E-rhBMP-2 were determined in the nanomolar range (10^–7^ to 10^–9^ M) and no significant difference on binding affinity was observed. Similar results were also reported in a molecular perspective study, claiming that N-glycans were not directly related to the rhBMP-2 epitope for binding BMP receptors (Kirsch et al. 2000).

Western blot and qRT-PCR analysis revealed that the binding of E-rhBMP-2 to BMP receptors could stimulate the osteogenic response via the classical Smad signaling pathway similar to that of C-rhBMP-2. Phosphorylation of R-Smads (Smad1/5/9) was detected and the intensity was increased up to 6 h in the C2C12 cells after treatment with E-rhBMP-2 and C-rhBMP-2. Since the phosphorylated R-Smads subsequently form a complex with Smad4 and are transferred into the nucleus, up- or downregulation of the specific genes should be observed (Wang et al. 2014; Pasero et al. 2012; Miyazono et al. 2010; Prashar et al. 2014). In this study, a significant increase in the mRNA expression level of the osteogenic marker genes, *Runx2* and *OCN,* was achieved indicating that E-rhBMP-2 was able to serve as an activator for osteogenic differentiation.

However, the intensity of R-Smads phosphorylation and the marker gene expression profile induced by E-rhBMP were clearly different from those of C-rhBMP. Smad1/5/9 phosphorylation at 3 h after C-rhBMP-2 treatment was higher than that on treatment with E-rhBMP-2. Based on these findings, it was determined that E-rhBMP-2 follows the signaling pathway regulated by Smads, but the initial intracytoplasmic phosphorylation rate is lower than that of C-rhBMP-2. In the case of *Runx2* and *OCN*, the gene expression level according to treatment time showed no statistically significant differences based on the type of rhBMP-2, but the expression level of *Runx2* at 18 h after C-rhBMP-2 treatment was higher than that on E-rhBMP-2 treatment. Similar to the Western blot results, these results demonstrate that the rate of osteogenic gene expression on E-rhBMP-2 treatment was slower than that on C-rhBMP-2 treatment.

ALP activity was measured to compare the potency of E-rhBMP-2 and C-rhBMP-2 in vitro. The relative potency of C-rhBMP-2 was approximately three times higher than that of E-rhBMP-2, which is consistent with the findings of previous studies (Fung et al. 2019; Suzuki et al. 2020). This difference in potency could be attributed to fast phosphorylation and the high expression levels of osteogenic genes found in the C-rhBMP-2 group from the phosphorylation results of R-Smads and the profiles of gene expression reported in a previous study (Yano et al. 2009). However, although the C2C12 cells were treated with high concentrations of E-rhBMP-2 and C-rhBMP-2, both groups showed similar levels of ALP activity, indicating that there was no difference between E-rhBMP-2 and C-rhBMP-2 with respect to efficacy. Many studies have reported that under in vivo conditions, when a sufficient amount of rhBMP-2 was exposed to a wound site with an effective carrier system like collagen or biodegradable polymer, E-rhBMP-2 and C-rhBMP-2 showed similar bone-inducing activities (Huh et al. 2011; Bessho et al. 2000).

The influence of E-rhBMP-2 and C-rhBMP-2 on overall gene expression was revealed by RNA-seq in a time-dependent manner. The number of DEGs between E-rhBMP-2 and C-rhBMP-2 groups at 3, 6, and 12 h after rhBMP-2 treatment was only two out of a total of 21,868 genes. On the contrary, 85 DEGs were found at 24 h after rhBMP-2 treatment; this indicates that the difference in gene expression profiles according to rhBMP-2 types occurred dramatically at 24 h post-treatment. Among the DEGs, several osteogenic markers were identified and classified into two groups depending on the response time. Immediate early response genes include inhibitors of differentiation or inhibitor of DNA binding 3(*Id3*), *OASIS*, and *TIEG,* and late response genes include type 1 collagen (*Col1*), osterix (*OSX*), osteoprotegerin (*OPG*), and osteopontin (*OPN*), along with *Runx2* and *OCN* (Miyazono et al. 2010; Sun et al. 2015).

The expression profiles of nine typical osteogenic marker genes were similar between the E-rhBMP-2 and C-rhBMP-2 groups. However, the level of gene expression at each time point was higher in the C-rhBMP-2 group compared to that in the E-rhBMP-2 group. In particular, the expression level of *OASIS* at 24 h after C-rhBMP-2 treatment was approximately four times higher than that in the E-rhBMP-2 group. *OASIS* is a transcription factor that activates the expression of type 1 collagen during osteogenesis (Kondo et al. 2012); therefore, the expression level of *Col1* at 24 h was higher in the C-rhBMP-2 group compared to that in the E-rhBMP-2 group. It is believed that this difference in the expression level of immediate-early response genes has been affected by intracytoplasmic phosphorylation rate, as confirmed by western blot analysis. Similarly, rapid intracytoplasmic phosphorylation rate determined in C-rhBMP-2 was related to the relatively high expression of osteogenic markers genes like late response osteogenic marker genes *OCN* and *OPG* in C-rhBMP-2 as compared to E-rhBMP-2, as confirmed by qPCR and RNA-seq analysis.

In Conclusion, the biochemical analysis confirmed that the osteogenic signaling pathway induced by E-rhBMP-2 or C-rhBMP-2 followed the Smad signaling pathway. Moreover, the difference in potency found between E-rhBMP-2 and C-rhBMP-2 could be attributed to the differences in intracellular phosphorylation and transcription rate of osteogenic genes, rather than the interaction between rhBMP-2 proteins and BMP receptors. As this study focused on the *in-vitro* results, we were unable to describe the signal pathway of E-rhBMP-2 during osteogenesis *in-vivo*. Despite this limitation, our study contributes to enhancing the understanding of the mechanism of E-rhBMP-2-induced osteogenesis and enables the application of E-rhBMP-2 in the field of bone regeneration medicine.

## Data Availability

RNA-seq raw data from this study have been deposited in the NCBI Short Read Archive under the accession number PRJNA812069.

## Abbreviations

ALP: Alkaline phosphatase
BMP: Bone morphogenetic protein
BMPR1A: BMP receptor type 1A
BMPR1B: BMP receptor type 1
BMPR2: BMP receptor type 2
BS: Bovine serum
CHO: Chinese hamster ovary
*Col1*: Type 1 collagen
C-rhBMP-2: Chinese hamster ovary cell-derived rhBMP-2
DEGs: Differentially expressed genes
DMEM: Dulbecco’s modified Eagle’s medium
E-rhBMP-2: *E. coli*-derived rhBMP-2
FBS: Fetal bovine serum
*K*_*D*_: Equilibrium dissociation rate constant
NGS: Next-generation sequencing
*OASIS*: Old astrocyte specifically induced substance
*OCN*: Osteocalcin
*OPG*: Osteoprotegerin
*OPN*: Osteopontin
*OSX*: Osterix
PS: Penicillin–streptomycin
rhBMP-2: Recombinant human BMP-2
RNA-seq: RNA sequencing
R-Smads: Receptor-regulated Smads
*Runx2*: Runt-related transcription factor 2
SD: Standard deviation
TGF-β: Transforming growth factor-β
*TIEG1*: TGF-β-inducible early gene
